# Associations Between Serum Osteocalcin and Metabolic, Nutritional, and Hormonal Status in Male Patients with Chronic Kidney Disease: A Cross-Sectional Study

**DOI:** 10.3390/biomedicines14102309

**Published:** 2026-10-12

**Authors:** Magdalena Markowska, Arkadiusz Lubas, Aleksandra Rymarz, Stanisław Niemczyk, Katarzyna Romejko

**Affiliations:** Department of Internal Diseases, Nephrology and Dialysis, Military Institute of Medicine—National Research Institute, 128 Szaserów Street, 04-141 Warsaw, Polandkromejko@wim.mil.pl (K.R.)

**Keywords:** chronic kidney disease, osteocalcin, biomarker, cross-sectional study

## Abstract

**Background:** Osteocalcin (OC) is a protein synthesized by osteoblasts and is involved in bone formation. OC also participates in the regulation of metabolic processes. Serum OC concentrations rise with kidney function decrease. The aim of this study was to evaluate the associations between serum OC concentrations and laboratory parameters reflecting metabolic, nutritional and hormonal status, as well as anthropometric and body composition measurements in patients with eGFR (estimated Glomerular Filtration Rate) < 60 mL/min/1.73 m^2^. **Methods:** The study sample consisted of 144 men: 98 patients with eGFR < 60 mL/min/1.73 m^2^ (the study group) and 46 individuals with eGFR ≥ 60 mL/min/1.73 m^2^ (the control group). Body composition parameters were estimated with the use of bioimpedance spectroscopy (BIS). Laboratory measurements, including serum OC were performed. **Results:** In univariable regression analysis we observed a negative association between OC and parameters related to adipose tissue and a positive relationship with variables reflecting fluid overload in the study group. Serum laboratory measurements showed that plasma OC was positively associated with serum creatinine and parathyroid hormone (PTH) and negatively with plasma hemoglobin, insulin, vitamin D and HOMA-IR (Homeostatic Model Assessment of Insulin Resistance). Multivariable backward regression analysis showed a negative association between OC and fat tissue index (FTI) and a positive relationship between OC and relative overhydration (Rel OH) and PTH in men with eGFR < 60 mL/min/1.73 m^2^. **Conclusions:** Our results suggest that in men with CKD (Chronic Kidney Disease) and eGFR < 60 mL/min/1.73 m^2^ not receiving dialysis, serum OC may be associated with metabolic, nutritional, and hormonal disturbances. The observed association between OC and parameters indicative of fluid overload also suggests a potential role of serum OC as a marker of overhydration in this population.

## 1. Introduction

Osteocalcin (OC) is a 46-amino acid protein synthesized by osteoblasts during bone formation processes [1]. OC plays a crucial role in the development and regeneration processes of the skeleton [2]. Directly after its synthesis, OC undergoes triple γ-carboxylation, which enhances its affinity for hydroxyapatite crystals, facilitating its incorporation into the bone matrix. An acidic environment generated by osteoclasts during bone resorption reverses these chemical reactions. After decarboxylation, OC loses its ability to bind with bone tissue and is released into the bloodstream, which results in its increased serum concentrations [3,4]. The undercarboxylated form of OC (ucOC) was observed to be negatively associated with bone quality [5]. Higher serum OC concentrations were observed in patients with osteoporosis and osteopenia [6,7,8,9,10,11]. Also, OC was identified as an independent predictor of osteoporosis severity [10,12].

Chronic kidney disease—mineral and bone disorder (CKD-MBD) is a common complication in patients with chronic kidney disease (CKD). Recent data published in Kidney International Reports indicates that this complication represents one of the most clinically significant disorders affecting the global CKD population, currently estimated at 673.7 million individuals [13]. Despite the consensus that CKD-MBD is a common complication, contemporary studies emphasize that its true incidence remains underestimated [14]. CKD-MBD is characterized by disturbances in calcium and phosphate metabolism, vitamin D deficiency, secondary hyperparathyroidism, impaired bone turnover and mineralization, as well as enhanced vascular calcification [12,15,16]. OC was found to reflect altered bone turnover in CKD-MBD. Its serum concentrations were positively associated with plasma levels of parathyroid hormone (PTH), suggesting that OC could serve as an indicator of bone turnover in CKD patients [12]. Additionally, in CKD population OC was considered as a useful marker in the diagnostic process of osteopenia and osteoporosis [17]. In pediatric CKD patients, undercarboxylated OC was a predictor of bone fractures [18]. The latest KDIGO (Kidney Disease: Improving Global Outcomes) guidelines highlight the vascular and soft tissue calcification associated with CKD-MBD, emphasizing its critical role in dramatically increasing cardiovascular mortality among patients with CKD [19]. Previous studies have suggested an association between serum OC levels and vascular stiffness [20,21,22]. Patients with CKD-MBD need a comprehensive, individualized management strategy delivered by multidisciplinary teams [23]. However, some therapeutic interventions aimed at improving bone metabolism may paradoxically accelerate vascular calcification, thereby worsening the overall cardiovascular risk profile in this vulnerable patient population [24,25].

The coexistence of CKD and impaired glucose metabolism is highly prevalent in clinical practice [26,27,28,29]. Furthermore, a comprehensive meta-analysis involving 2.8 million individuals demonstrated that prediabetes is significantly associated with an increased risk of developing CKD [27]. Insulin resistance and diabetes significantly worsen the prognosis of patients with CKD [28,29]. OC was found to be involved in metabolic processes. After binding insulin to its receptor (InsR), a feedback loop is initiated, resulting in increased OC secretion. The circulating form of OC acts on both pancreatic and adipose tissue cells, thereby modulating metabolic homeostasis [30]. By increasing adiponectin synthesis in adipocytes, OC improves insulin sensitivity [31]. OC also directly stimulates pancreatic β-cells, leading to upregulation of insulin secretion. Comparative analyses of serum OC concentrations between healthy individuals and patients with insulin resistance identified OC as a predictor of insulin sensitivity [32]. Serum OC was further established as an independent negative determinant of plasma glucose concentrations [33]. Several studies reported negative associations between plasma OC levels and hemoglobin A1c (HgbA1c) [34,35]. Additionally, decreased serum concentrations of both carboxylated and undercarboxylated forms of OC, along with a higher proportion of the carboxylated variant, were associated with an increased risk of metabolic syndrome [30,31].

Malnutrition is a common complication of CKD. The prevalence of malnutrition increases progressively with the advancement of CKD and also depends on ethnic background. While malnutrition affects approximately 18% of patients with CKD, its prevalence may reach up to 47% among individuals of African descent [36,37,38]. The development of nutritional deterioration in CKD is multifactorial and involves reduced dietary intake, chronic inflammation, metabolic acidosis, hormonal disturbances and increased protein catabolism, all of which contribute to progressive body weight loss and reduction in nutritional reserves [39]. One of the component of this process is protein-energy wasting (PEW), characterized by the loss of body protein and energy stores, including both skeletal muscle and adipose tissue [40]. PEW frequently coexists with chronic inflammation and accelerated atherosclerosis, forming the malnutrition–inflammation–atherosclerosis (MIA) syndrome, which is strongly associated with increased cardiovascular morbidity and mortality [41]. Furthermore, CKD patients commonly develop sarcopenia, manifested by loss of skeletal muscle mass and muscle strength, often accompanied by unintentional weight loss and functional decline [42]. Progressive reduction in body weight and skeletal muscle mass in CKD population is associated with frailty, poor quality of life, increased hospitalization rates and unfavorable clinical outcomes. Consequently, malnutrition and weight loss in CKD are recognized as major predictors of poor prognosis and mortality [43]. Therefore, the diagnostic component, which comprises assessing nutritional status using standardized measures, remains essential in patients with CKD [44,45,46]. Growing evidence suggests that OC is also associated with nutritional status. Previous studies demonstrated significantly lower serum OC concentrations in malnourished children compared to healthy controls [47]. Moreover, OC levels were observed to fluctuate in response to short-term changes in food intake, indicating that this bone-derived protein may serve as a sensitive biomarker of systemic nutritional status, particularly protein-energy balance [48].

The primary objective of this study was to investigate the association between serum OC concentrations and CKD complications in men with an eGFR (estimated Glomerular Filtration Rate) < 60 mL/min/1.73 m^2^ not treated with dialysis. The secondary objectives were to compare the relationship between plasma OC and various laboratory parameters reflecting metabolic, nutritional, and hormonal status, as well as anthropometric and body composition measurements, between patients with CKD and eGFR < 60 mL/min/1.73 m^2^, and individuals with eGFR ≥ 60 mL/min/1.73 m^2^.

## 2. Materials and Methods

### 2.1. Patients

One hundred and forty-four male patients were included in the study. Participants were classified into two groups according to their renal function: 98 patients with eGFR < 60 mL/min/1.73 m^2^ (the study group) and 46 individuals with eGFR ≥ 60 mL/min/1.73 m^2^ (the control group), which included participants with preserved kidney function. The study group consisted of 65 patients with eGFR 30–59 mL/min/1.73 m^2^, 24 patients with eGFR 15–29 mL/min/1.73 m^2^, and 9 patients with eGFR < 15 mL/min/1.73 m^2^. Participants from the study group were recruited from patients attending the Nephrology Outpatient Clinic for routine check-up. Men from the control group were enrolled from the Internal Medicine Department. The inclusion criteria was age between 18 and 80 years for both groups, eGFR < 60 mL/min/1.73 m^2^ for the study group and eGFR ≥ 60 mL/min/1.73 m^2^ for the control group. The exclusion criteria consisted of renal replacement therapy or the need for this form of treatment within the following three months, clinical signs of infection, and the presence of metallic implants. Moreover, individuals with a history of CKD or clinical evidence of CKD were excluded from the control group during the selection process. The study protocol was approved by the local ethics committee (Bioethics Committee at the Military Institute of Medicine, IRB approval number 120/WIM/2018, dated 22 August 2018). All participants provided written informed consent to participate in the study.

### 2.2. Body Composition Parameters

Body composition parameters were assessed by bioelectrical impedance spectroscopy (BIS) with the use of Body Composition Monitor (BCM, Fresenius Medical Care, Bad Homburg, Germany). Participants were examined in the supine position after a five-minute rest, with electrodes placed on one hand and one foot in a standard tetrapolar configuration. The analysis provided estimates of lean tissue mass (LTM), relative LTM (Rel LTM), lean tissue index (LTI), fat mass (Fat), relative fat mass (Rel fat), fat tissue index (FTI), adipose tissue mass (ATM), overhydration (OH), relative OH (Rel OH), total body water (TBW), extracellular water (ECW) and intracellular water (ICW). LTI was measured as LTM in kilograms and FTI was calculated as Fat in kilograms, both divided by height in square meters.

### 2.3. Laboratory Measurements

Blood samples were collected after an overnight fast. Serum samples for measuring OC were kept frozen at −80 °C. OC concentrations were assessed using a Luminex MAGPIX multiplex immunoassay (Luminex Corporation, Austin, TX, USA). Plasma creatinine concentrations were measured using the Jaffe method (Gen.2; Roche Diagnostics GmbH, Risch-Rotkreuz, Switzerland). Serum albumin levels were determined by the a bromocresol green colorimetric assay with the use of a BCP Albumin Assay Kit (Roche Diagnostics GmbH, Risch-Rotkreuz, Switzerland). C-reactive protein (CRP) serum concentrations were assessed by an immunoturbidimetric assay on a Cobas c 503 PRO analyzer (Roche Diagnostics, Mannheim, Germany). Hemoglobin concentrations were measured using the sodium lauryl sulfate method on a Sysmex XN-1000 hematology analyzer (Sysmex Corporation, Kobe, Japan). Plasma glucose levels were quantified using a hexokinase enzymatic assay on a Cobas c 503 PRO analyzer (Roche Diagnostics, Mannheim, Germany). Serum insulin, peptide-C, PTH, and vitamin D concentrations were assessed using an electrochemiluminescence immunoassay on a Cobas e411 analyzer (Roche Diagnostics, Mannheim, Germany). HgbA1c was measured with the use of the high-performance liquid chromatography method on a Tosoh HLC-723 G8 analyzer (Tosoh Bioscience, Tokyo, Japan). Triglycerides, total cholesterol (TC), low-density lipoprotein (LDL) cholesterol, and high-density lipoprotein (HDL) cholesterol were determined using the enzymatic colorimetric methods, with a homogeneous assay applied for HDL and LDL cholesterol on a Cobas c 503 PRO analyzer (Roche Diagnostics, Mannheim, Germany).

### 2.4. The Assessment of eGFR

The estimated glomerular filtration rate (eGFR) was calculated using the 2021 Chronic Kidney Disease Epidemiology Collaboration (CKD-EPI) creatinine equation:

eGFRcr = 142 × min (Scr/κ, 1) α × max (Scr/κ, 1) − 1200 × 0.9938 Age

Scr = serum creatinine in mg/dL; κ = 0.9; α = −0.302; min (Scr/κ, 1); and max (Scr/κ, 1) represent the smaller and larger of Scr/κ or 1.0, respectively; Age = age in years.

### 2.5. Statistical Analysis

Results were presented as mean with standard deviation (SD) and median with interquartile range (IQR). Compliance with a normal distribution was assessed using the Shapiro–Wilk test. Pearson or Spearman correlation tests were used for correlation assessment depending on the concordance with normal distribution. Parametric, normally distributed independent data were compared with a *t*-test; otherwise, with the Mann–Whitney U test. Univariate linear regression analysis was performed to test the association with osteocalcin. In a nonlinear relation, variables were transformed by logarithmization. Then, a multivariable backward regression analysis was performed for significantly associated representative variables. If several related variables reflecting the same phenomenon were significantly associated with OC, the variable with the highest loading was selected for the regression model. Missing data were not supplemented. For each test, *p* < 0.05 was considered significant. For statistical analysis, Statistica v.13.3 software (StatSoft Inc., TIBCO Software Inc., Greenwood Village, CO, USA) was used.

## 3. Results

Patients with eGFR < 60 mL/min/1.73 m^2^ were older compared to individuals with eGFR ≥ 60 mL/min/1.73 m^2^ (*p* < 0.001). The groups also differed in terms of systolic (SBP) and diastolic blood pressure (DBP), which were significantly higher in the study group (*p* = 0.005 and *p* < 0.001, respectively). Based on body composition parameters, participants with eGFR ≥ 60 mL/min/1.73 m^2^ had lower LTI (*p* = 0.038). In laboratory measurements, serum OC was significantly higher in the study group (*p* = 0.002). Serum CRP and hemoglobin levels were lower in individuals with eGFR < 60 mL/min/1.73 m^2^ (*p* < 0.001 and *p* < 0.001, respectively). Patients with eGFR < 60 mL/min/1.73 m^2^ had higher serum peptide C (*p* < 0.001), as well as HgbA1c (*p* = 0.007). Men in the study group had lower mean serum TC (*p* = 0.030) but higher plasma triglycerides concentrations (*p* = 0.025). PTH concentrations were higher in men with eGFR < 60 mL/min/1.73 m^2^ (*p* < 0.001) (Table 1).

Serum OC correlated negatively with age in the study group (*p* = 0.041) and positively with SBP in men with eGFR < 60 mL/min/1.73 m^2^ and in the entire cohort (*p* = 0.011 and *p* = 0.028, respectively). OC was negatively associated with weight in patients with eGFR ≥ 60 mL/min/1.73 m^2^ (*p* = 0.016) and in all participants (*p* = 0.024). The significant negative association between plasma OC and BMI was observed in both groups (*p* = 0.004 in the study group and *p* = 0.022 in the control group) and remained significant when the two groups were analyzed together (*p* < 0.001).

Regarding body composition parameters, serum OC correlated positively with Rel LTM in men with eGFR < 60 mL/min/1.73 m^2^ (*p* = 0.033) and also in all participants (*p* = 0.013). OC was related negatively with all parameters associated with fat tissue, such as Fat, Rel fat, FTI, and ATM in the study group (*p* = 0.005, *p* = 0.007, *p* < 0.001, and *p* = 0.005, respectively) and in the full sample (*p* = 0.003, *p* = 0.005, *p* < 0.001, and *p* = 0.003, respectively). Serum OC was also positively associated with Rel OH in patients with eGFR < 60 mL/min/1.73 m^2^ (*p* = 0.005) and negatively with ECW in individuals with eGFR ≥ 60 mL/min/1.73 m^2^ (*p* = 0.012).

In laboratory measurements, OC was positively associated with serum creatinine in patients with eGFR < 60 mL/min/1.73 m^2^ (*p* < 0.001) and in the study cohort as a whole (*p* < 0.001), and negatively with eGFR value (*p* < 0.001 and *p* < 0.001, respectively). OC negatively correlated with serum albumin in the study group (*p* < 0.001) and all participants (*p* = 0.029); however, in individuals with eGFR ≥ 60 mL/min/1.73 m^2^, this association remained positive (*p* = 0.019). We also observed a negative relationship between OC and hemoglobin in men with eGFR < 60 mL/min/1.73 m^2^ (*p* = 0.002) and in the entire group (*p* = 0.001). Plasma OC negatively correlated with insulin and HOMA-IR in the study group (*p* = 0.002 and *p* = 0.029, respectively) and in all participants combined (*p* = 0.003 and *p* = 0.031, respectively), and with triglycerides in men with eGFR ≥ 60 mL/min/1.73 m^2^ (*p* = 0.018). Regarding PTH, we observed a positive association with serum OC in patients with eGFR < 60 mL/min/1.73 m^2^ (*p* < 0.001) and in all participants (*p* < 0.001) (Table 2).

**Figure 1:**
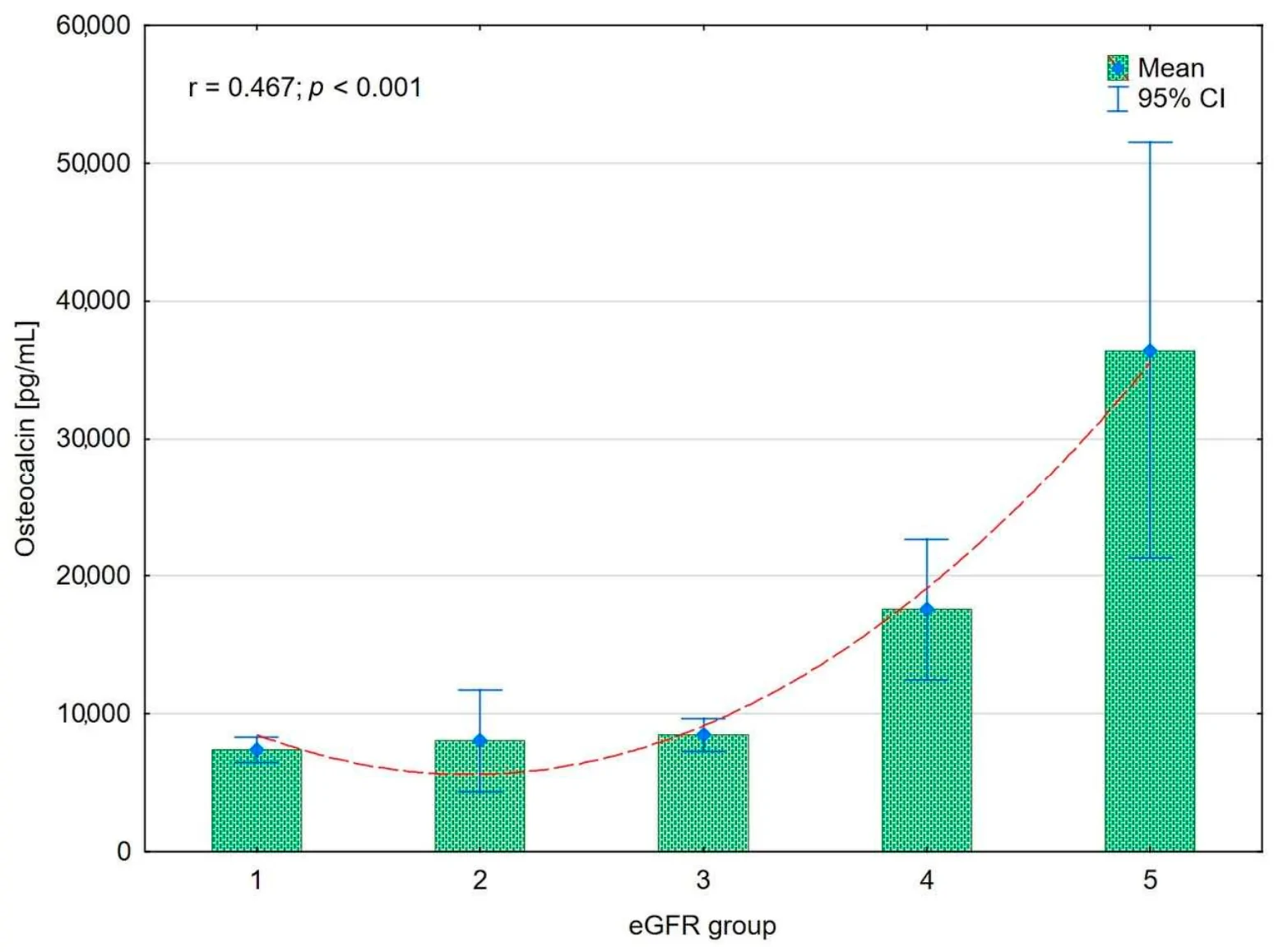
The association of serum osteocalcin with patient groups assessed by eGFR value. Dashed red line—polynomial regression line for considered variables.

When we classified the whole study sample according to eGFR assessed with the use of CKD-EPI into 5 groups—group 1 (n = 30) with eGFR ≥ 90 mL/min/1.73 m^2^, group 2 (n = 16) with eGFR 60–89 mL/min/1.73 m^2^, group 3 (n = 65) with eGFR 30–59 mL/min/1.73 m^2^, group 4 (n = 24) with eGFR 15–29 mL/min/1.73 m^2^ and group 5 (n = 9) with eGFR < 15 mL/min/1.73 m^2^ we observed that serum OC did not differ between individuals assigned to groups 1, 2 and 3, and then increased with the subsequent decline of eGFR (r = 0.467, *p* < 0.001) (Figure 1).

**Figure 2:**
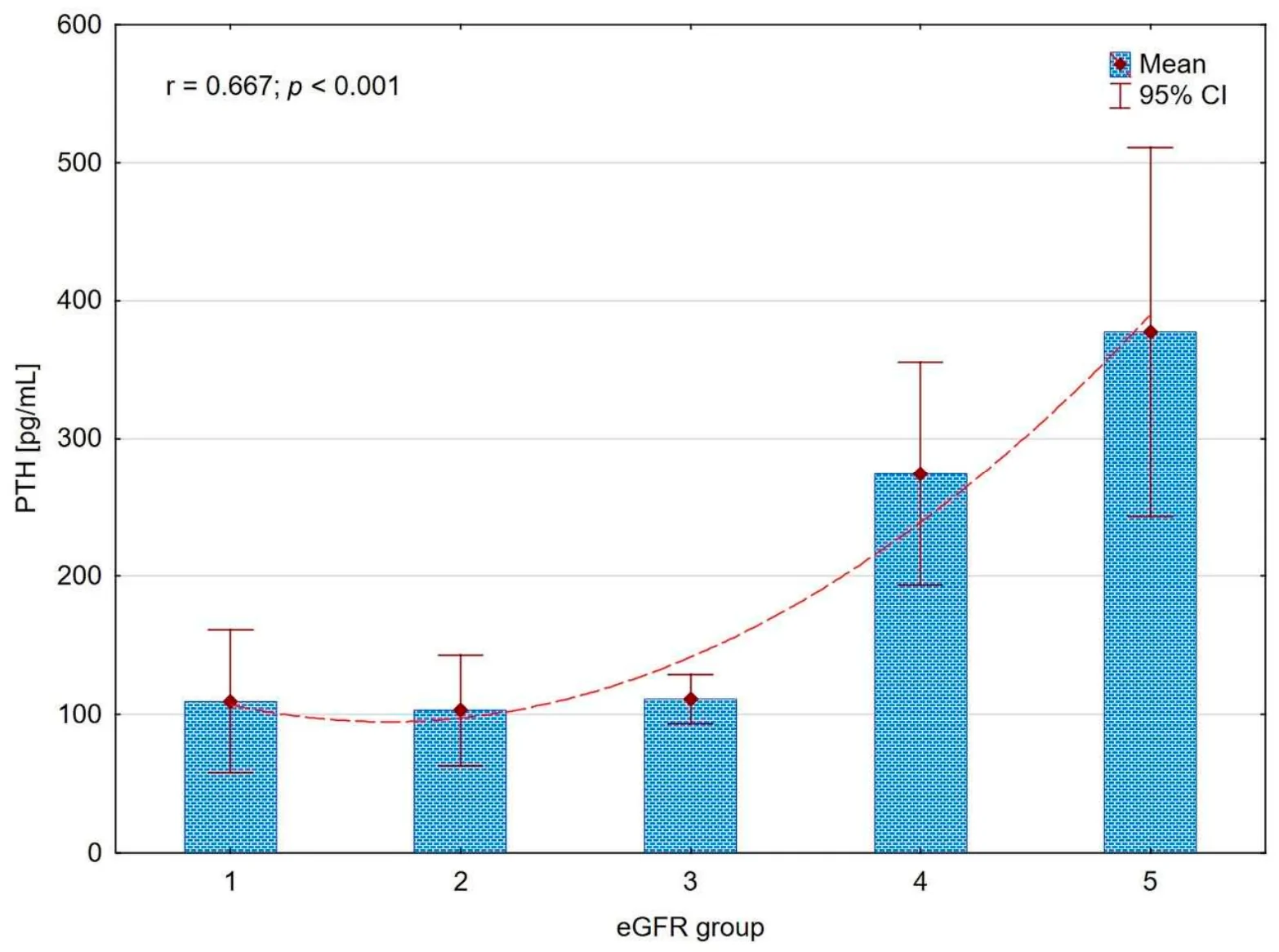
The association between serum PTH and patient groups assessed by eGFR. Dashed red line—polynomial regression line for considered variables.

Serum PTH concentrations were similar in groups 1, 2 and 3, and increased in patients with eGFR ≤ 29 mL/min/1.73 m^2^ (r = 0.667, *p* < 0.001) (Figure 2).

Univariable regression analysis showed a positive association between serum OC and SBP in the study group (*p* < 0.001) and in all participants combined (*p* < 0.001). Based on body composition parameters plasma OC was positively associated with Rel LTM in patients with eGFR < 60 mL/min/1.73 m^2^ (*p* = 0.026) and in the entire cohort (*p* = 0.016). In the study group and all participants, we observed a negative association between OC and parameters related to fat tissue such as Fat, Rel fat, FTI, and ATM (*p* = 0.008, *p* = 0.002, *p* = 0.003, *p* = 0.008, respectively, for the study group and *p* = 0.006, *p* = 0.001, *p* = 0.003, *p* = 0.006, respectively, for the full sample). Depending on hydration status, plasma OC was positively associated with OH and Rel OH in the study group (*p* < 0.001, *p* < 0.001, respectively), and negatively with ECW in men with eGFR ≥ 60 mL/min/1.73 m^2^ (*p* = 0.040).

Laboratory parameters showed a positive association between OC and serum creatinine in the study group (*p* < 0.001) and all participants (*p* < 0.001), and a negative relationship between OC and eGFR in these groups (*p* < 0.001, *p* < 0.001, respectively). Plasma albumin was positively related to OC in the control group only (*p* = 0.016). We found a negative association between OC and serum hemoglobin in patients with eGFR < 60 mL/min/1.73 m^2^ (*p* < 0.001) and in the entire cohort (*p* < 0.001). A negative relationship between OC and insulin as well as HOMA-IR was also observed in the study group (*p* = 0.039 and *p* = 0.039) and in all participants (*p* = 0.031 and *p* = 0.031). Additionally, OC was negatively associated with triglycerides in the control group (*p* = 0.020). In univariate regression analysis, we also found a positive relationship between OC and PTH in patients with eGFR < 60 mL/min/1.73 m^2^ (*p* < 0.001) and in the entire cohort (*p* < 0.001), and a negative association between OC and vitamin D in the study group (*p* = 0.013) (Table 3).

In multivariable backward regression analysis the model for the total cohort included SBP, FTI, eGFR, hemoglobin, log-transformed insulin and PTH. The model for the control group included ECW, albumin and TG. The model for the CKD group included SBP, FTI, Rel OH, eGFR, hemoglobin, log-transformed insulin, PTH and log-transformed vitamin D. In the study group, multivariable regression analysis revealed that Rel OH (*p* = 0.014), FTI (*p* = 0.006), and PTH (*p* = 0.007) were independently associated with OC (R^2^ = 0.508, *p* < 0.001). Similar results were obtained in backward regression analysis, in which OC was independently positively associated with Rel OH and PTH, and negatively related with FTI (*p* = 0.001, *p* < 0.001, *p* = 0.002, respectively), explaining 47.0% of its variability (R^2^ = 0.470, *p* < 0.001). In the control group, multivariable regression analysis showed that only albumin concentration (*p* = 0.047) was significantly related to OC (R^2^ = 0.223, *p* = 0.017). By contrast, backward regression analysis showed that OC was independently positively associated with albumin and negatively associated with TG (*p* = 0.034, *p* = 0.045, respectively), explaining 20.0% of its variability (R^2^ = 0.200, *p* = 0.010). In the total cohort, multivariable regression analysis revealed that only FTI (*p* = 0.009) and PTH (*p* < 0.001) were independently associated with OC (R^2^ = 0.426, *p* < 0.001). However, in backward elimination, OC was independently negatively associated with FTI and hemoglobin (*p* = 0.002, *p* < 0.001, respectively), and positively associated with PTH (*p* = 0.022), explaining 41.8% of its variability (R^2^ = 0.418, *p* < 0.001) (Table 4).

## 4. Discussion

In our study, we found significant associations between OC and parameters reflecting metabolic, nutritional and hormonal disturbances in CKD men with eGFR < 60 mL/min/1.73 m^2^ not treated with dialysis. We also observed a positive relationship between OC and fluid overload in this population.

### 4.1. Serum Osteocalcin and Anthropometric Parameters

In our study, serum OC concentrations significantly correlated with anthropometric parameters. Higher plasma OC levels in the study group were associated with lower BMI, higher Rel LTM, and lower parameters related to fat tissue, such as Fat, Rel fat, FTI, and ATM. Additionally, serum OC concentrations positively correlated with OH and Rel OH. A negative relationship with Fat, Rel fat, FTI, and ATM as well as a positive relationship with Rel LTM, OH and Rel OH remained significant when assessed by univariable analysis. After multivariable adjustment, OC remained independently negatively associated with FTI and positively with Rel OH. In the control group, plasma OC levels negatively correlated with weight and BMI. Also, a negative correlation between OC and ECW was found, which was confirmed as well in univariable analysis, indicating an inverse relationship between OC and fluid overload in these two groups.

The results of our study show that higher serum OC concentrations are associated with a less favorable metabolic and nutritional profile in patients with eGFR < 60 mL/min/1.73 m^2^ compared to individuals with eGFR ≥ 60 mL/min/1.73 m^2^. According to the reverse epidemiology phenomenon, or obesity paradox in CKD, overweight patients (BMI value from 25.0 to 29.9 kg/m^2^) or those with obesity class 1 (BMI value from 30.0 to 34.9 kg/m^2^) or class 2 (BMI value from 35.0 to 39.9 kg/m^2^) have lower mortality compared to individuals with correct BMI (18.5–24.9 kg/m^2^) [49]. Additionally, obesity class 3 (BMI value ≥ 40.0 kg/m^2^) was not associated with higher mortality rate among CKD patients [50]. Patients treated with dialysis with BMI above 30 kg/m^2^ also had lower all-cause mortality [51]. In contrast, in the general population, overweight or obese individuals have higher mortality compared to those with normal BMI [51]. However, in CKD, similarly to the general population, malnutrition (BMI value < 18.5 kg/m^2^) was also associated with higher mortality [52,53,54]. Increased BMI is related to greater adiposity. In our study, the negative correlation between serum OC and BMI, and the adverse relationship between OC and parameters associated with fat mass in patients with eGFR < 60 mL/min/1.73 m^2^ indicate that higher serum OC concentrations may be associated with lower body and adipose tissue mass, which, taking into account the obesity paradox in CKD, might be related to adverse outcomes. However, the relationships between body weight, body composition, and clinical status are complex and remain incompletely understood, particularly in the context of CKD, where multiple metabolic, hormonal, and clinical factors may interact, and therefore, higher serum OC concentrations should not be interpreted unequivocally as indicative of an unfavorable clinical phenotype. Since the negative association between OC and FTI remained significant in multivariable analysis, we may conclude that OC may serve as a potential biomarker of nutritional and metabolic disturbances in CKD patients. In the group of patients with eGFR ≥ 60 mL/min/1.73 m^2^, serum OC also negatively correlated with BMI and weight. Some other studies revealed similar results [55,56]. A systematic review and meta-analysis of 51 observational studies confirmed a significant relationship between serum OC concentrations and BMI. Both total OC and undercarboxylated OC were inversely associated with BMI and body fat percentage [55]. The preclinical study of Lee involving animals found that the absence of OC led to obesity. Furthermore, an atypical accumulation of visceral fat was observed in OC deficiency. Not only was the overall fat mass higher, but also the number of adipocytes was increased [57,58,59]. The study of Nowicki also showed that circulating undercarboxylated OC levels were inversely associated with BMI, total body fat, visceral adipose tissue, and hepatic fat accumulation, suggesting its protective role against obesity in the general population [1]. However, taking into account the obesity paradox in CKD, this favorable role of OC in the general population may exert an opposite effect in patients with kidney function decrease.

Several studies have demonstrated a positive association between OC levels and skeletal muscle mass. Muscle atrophy is associated with increased morbidity, higher number of hospitalizations and elevated mortality rate, not only in the general elderly population, but also in CKD patients [60,61,62]. A prospective observational study of Xiang demonstrated that OC may contribute to the maintenance of skeletal muscle mass during weight loss in overweight and obese men without metabolic syndrome [63]. In postmenopausal women, elevated serum OC concentrations were also found to be associated with increased muscle mass [7]. Consistent with previous observations, Smith showed that higher circulating OC concentrations were related to more favorable body composition, with lower fat mass and higher skeletal muscle mass, which suggests a potential protective role of OC in age-related muscle decline [64]. A recent meta-analysis demonstrated a significant positive association between circulating total OC and undercarboxylated OC concentrations, and the percentage of lean tissue mass. The subgroup analyses indicated a stronger association between OC levels and muscle mass—related parameters in men, suggesting potential sex-specific differences in the relationship between OC and muscle mass [65]. In our study, the significant positive correlation between OC and Rel LTM in patients with eGFR < 60 mL/min/1.73 m^2^, confirmed by univariable analysis, may indicate a potential association between higher serum OC concentrations and preserved lean tissue in patients with CKD. However, it needs to be confirmed in future studies involving patients with kidney function decrease.

Fluid overload is a serious complication of CKD. Excess fluid accumulation was identified as an independent predictor of adverse outcomes in patients with advanced CKD, being associated with an increased risk of all-cause mortality and cardiovascular morbidity in this group [66,67,68]. Early diagnosis of fluid imbalance may therefore constitute an important component of multidisciplinary CKD management [69,70]. Borges demonstrated that the OH index was the bioimpedance—derived parameter most strongly associated with clinical markers of hydration status, including blood pressure, interdialytic weight gain, and N-terminal pro-B-type natriuretic peptide (NT-proBNP), supporting its utility for assessing volume status [71]. In currently available literature, clinical studies did not directly investigate the relationship between fluid overload and serum OC concentrations. However, Rel OH was found to be associated with malnutrition. Antlanger assessed hydration status in a cohort of 244 patients receiving hemodialysis (HD), which was evaluated using BIS. Patients with fluid overload had lower BMI and reduced serum albumin concentrations, both indicative of poor nutritional status [59]. Comparable observations were reported in HD pediatric population, which revealed a significant inverse correlation between nutritional status, as measured by the normalized protein catabolic rate (nPCR), and hydration status, assessed using the Refill Index (RI) [72]. Evidence also indicated that any degree of Rel OH was significantly associated with elevated long-term mortality among non-dialysis CKD patients with eGFR below 29 mL/min/1.73 m^2^ [73]. Taken together, these observations suggest that fluid overload in CKD may reflect an overall deterioration in clinical condition, including impaired nutritional status. Thus, the positive significant association between OC and parameters indicative of fluid overload observed in patients with eGFR < 60 mL/min/1.73 m^2^ in our study suggests a potential relationship of OC with both fluid status and nutritional disturbances in patients with CKD. Further studies are needed to determine whether OC may have value as a biomarker of these conditions.

### 4.2. Serum Osteocalcin and Biochemical Parameters

In our study, serum OC concentrations were associated with biochemical parameters reflecting metabolic or nutritional status. In the study group, a positive correlation was found between serum OC levels and PTH. Plasma OC was also negatively associated with serum albumin, and hemoglobin. These observations allow us to hypothesize that in patients with CKD, serum OC may be an indicator of major CKD-related complications, including anemia, malnutrition, and secondary hyperparathyroidism. However, these associations should be interpreted in the context of impaired renal function and CKD-MBD, as both reduced kidney function and disturbances in mineral and bone metabolism may affect circulating OC concentrations. Thus, serum OC should not be interpreted as an independent marker of these complications, but rather as a potential integrative indicator reflecting the complex metabolic, hormonal, and skeletal disturbances accompanying CKD. On the contrary, in the control group, serum OC concentrations correlated positively with plasma albumin and negatively with triglycerides, suggesting that in the population with eGFR ≥ 60 mL/min/1.73 m^2^, higher OC concentrations are associated with more beneficial metabolic status. In the univariable analysis, OC was negatively associated with hemoglobin and vitamin D, but positively associated with PTH in the study group. Conversely, in the control group, a positive correlation with albumin and negative with triglycerides were confirmed. Similar associations were observed in the multivariable analysis, where OC was positively related with PTH in the study group, whereas, in the control group, it was positively associated with albumin and negatively with triglyceride levels. The results confirm that OC may be associated with metabolic, nutritional and hormonal disturbances in CKD.

The relationship between OC and PTH in our study is consistent with the physiological association between bone turnover and PTH activity, and is supported by prior clinical research. The study of Chi, which included 95 patients with CKD stages G2–G5, found a positive relationship between OC and serum PTH concentrations. The multivariate regression analysis revealed that serum OC is an independent determinant of osteoporosis severity in CKD population. Based on these observations, the authors concluded that OC may serve as a marker of bone turnover in patients with CKD [12]. Osteoporosis and bone fractures are severe complications in individuals with advanced CKD [74,75,76]. However, the clinical assessment of osteoporosis in this population is often confounded, making it difficult to identify patients at the highest risk of bone fractures. Thus, reliable markers of impaired bone metabolism in CKD patients are needed, as they could facilitate the timely detection of osteoporosis, already present in the early stages of CKD, and enable the implementation of appropriate treatment and preventive strategies [77,78,79]. A recent study directly investigating OC in the context of CKD evaluated uncarboxylated OC as a potential predictor of bone fractures in children with CKD. The observations demonstrated that increased serum OC concentrations were associated with the presence of fractures [18]. Growing evidence indicate that elevated circulating OC concentrations are associated with reduced bone mineral density, particularly in conditions characterized by increased bone turnover. Therefore, OC may represent a valuable biochemical marker for evaluating bone metabolic activity and supporting the diagnosis, monitoring, and therapeutic management of osteoporosis [6,80]. However, current evidence remains insufficient to justify the use of OC as an independent predictor of fracture risk, CKD-MBD progression, or as a standalone biomarker for therapeutic decision-making. Therefore, OC concentrations should be interpreted alongside established markers of bone metabolism, such as PTH and bone-specific alkaline phosphatase, to provide a more comprehensive evaluation of skeletal status [81].

It should be noted, in the context of CKD-MBD, that alterations in OC levels may reflect not only disturbances in bone turnover but also broader endocrine and metabolic adaptations associated with CKD [1,80,82,83]. Karsenty et al. emphasized the role of OC in mediating bone-organ communication and whole-body homeostasis. It contributes to the regulation of energy metabolism, glucose homeostasis, steroidogenesis, male fertility, cognition, stress responses, and exercise capacity [82]. The study of Determe also highlighted the potential role of OC in mediating bone–organ crosstalk [83].

### 4.3. Serum Osteocalcin and Glucose Metabolism

In our study, negative correlations were observed between serum OC concentrations and plasma insulin and HOMA-IR, both in the study group and in the entire cohort. These negative associations were further confirmed by univariable regression analysis in both groups. However, neither of them remained significant in the multivariable analysis, suggesting that the observed correlations may be influenced by other clinical variables.

The associations between serum OC levels and glucose metabolism parameters observed in our study support the hypothesis that OC may be involved in the regulation of insulin sensitivity. This observation is consistent with previous studies demonstrating the involvement of OC in glucose and lipid metabolism, as well as its interaction with insulin and adipokine signaling pathways. Importantly, previous studies suggest that the involvement of OC in metabolic regulation may extend to patients with CKD [20,56,84]. In an animal model, Lee demonstrated that the absence of ucOC led to obesity and diabetes, accompanied by a reduced number of β-cells, impaired glucose tolerance, and increased insulin resistance. These findings led the authors to hypothesize that ucOC may enhance β-cell proliferation in the pancreas, as well as insulin and adiponectin expression in pancreatic islets and in adipose tissue [85]. Bilotta reported crosstalk between bone and energy metabolism in both in vitro models and humans. They found that circulating total OC was associated with enhanced insulin sensitivity in peripheral tissues [86]. It is important to note that this interaction appears to involve a bidirectional feedback loop, with OC influencing insulin action and insulin, in turn, stimulating OC synthesis upon binding to its receptor on osteoblasts [87,88]. Wei found that bone-specific insulin resistance disrupts systemic glucose homeostasis through the downregulation of ucOC activation. High-fat diet-induced reduction in skeletal insulin receptors decreased circulating active OC levels, creating a feedback loop that exacerbates systemic insulin resistance [88]. Furthermore, experimental evidence indicates that undercarboxylated OC stimulates glucagon-like peptide-1 (GLP-1) secretion from intestinal enteroendocrine L cells via GPRC6A signaling, thereby enhancing glucose-stimulated insulin secretion and improving glucose homeostasis [1,80,89,90]. Tiwari reported significantly lower circulating OC concentrations in patients with type 2 diabetes mellitus compared to healthy controls. Moreover, OC levels were inversely associated with glycated hemoglobin and fasting plasma glucose concentrations, suggesting that reduced OC levels may be associated with impaired glycemic control and increased insulin resistance. These observations confirm the potential role of OC as a bone-derived endocrine regulator involved in glucose homeostasis [91].

Overall, the negative associations between serum OC concentrations and insulin and HOMA-IR observed in our study correspond with previous evidence suggesting a potential link between OC and insulin sensitivity. However, the lack of independent associations in the multivariable analysis indicates that these relationships may be influenced by other clinical factors. The complex relationships observed between OC and metabolic parameters may reflect the broader interplay between energy homeostasis, insulin signaling, and tissue-specific metabolic regulation, rather than a direct association with a single metabolic pathway [92].

### 4.4. Clinical Implications and Perspectives for Practice

Malnutrition, reduced body mass, fluid overload, calcium–phosphate derangements with consequent impaired bone turnover, and insulin resistance are common complications of CKD. These disturbances are associated with numerous clinical complications, including cardiovascular disease, accelerated atherosclerosis, vascular calcification, anemia, elevated fracture risk, PEW and sarcopenia, which contribute to increased morbidity and mortality. Biomarkers of metabolic, hormonal, and nutritional disturbances may enable their early identification and allow for the implementation of timely preventive and therapeutic interventions.

Our observations suggest that serum OC may be associated with metabolic, nutritional, and hormonal disturbances in men with CKD not receiving dialysis. Given the multifactorial nature of CKD-related complications, including alterations in bone turnover, impaired glucose metabolism and changes in body composition, OC may provide additional information on the overall metabolic and nutritional profile. Although further prospective studies are required to establish its clinical utility, OC may have potential clinical relevance for identifying patients with metabolic, nutritional, and hormonal disturbances, and for improving the multidimensional assessment of CKD-related disturbances. The inclusion of OC measurements as part of a comprehensive CKD evaluation could potentially improve the characterization of disease-related abnormalities, particularly when interpreted together with established markers of mineral and bone metabolism, nutritional status, and metabolic control. More broadly, the clinical interpretation of circulating biomarkers requires consideration of their biological context and their relationship to measurable physiological or disease-related processes, rather than assuming that an observed association necessarily translates into diagnostic or prognostic utility [93].

In our study, we aimed to highlight metabolic differences between patients with CKD and individuals without kidney disease. In this context, OC seems to be interesting area for further investigation, given the growing evidence suggesting that it may exert pleiotropic effects extending beyond its traditional role in bone metabolism. Importantly, many of the available studies on OC in CKD has been generated in patients receiving HD, whereas data from patients with non-dialysis-dependent CKD remain relatively limited. Further prospective studies are needed to establish whether OC may serve as a useful parameter for risk stratification and contribute to optimizing individualized clinical management in patients with CKD.

#### The Study Limitations

The present study has several limitations. The first is the relatively small study population. Expanding the study cohort would enable a more detailed and more comprehensive analysis. Greater statistical validity could be provided to the correlation analyses. Furthermore, despite the large number of correlations, including variables that were closely related, we did not adjust for multiple testing. However, correlation analysis was only a preliminary step in hierarchical variable selection, and further tests verified corre-lations, which justifies this approach. Moreover, the backward multivariate regression analysis we used can eliminate variables with the least significance and relevance to the variable being modeled. Because CKD-EPI eGFR included age, we did not perform addi-tional age adjustments in multivariable analyses. On the other hand, age was not associ-ated with OC in univariable regression analysis in the considered groups. Although both groups included predominantly middle-aged to older men, age may have influenced some of the metabolic, body composition, and hormonal parameters assessed. Therefore, the possibility of residual confounding related to age cannot be completely excluded and should be considered when interpreting the findings. Also, the CKD patients and the control group differed in age. Additionally, only male participants were included. This strategy was adopted to obtain a more homogeneous study population with respect to body composition and hormonal profile. As the study included only male participants, the findings should not be directly extrapolated to women due to potential sex-related differences in bone metabolism, body composition, hormonal status, and hydration parameters. Future studies including both sexes are needed to determine whether the observed associations differ between men and women. Furthermore, the study population was limited to Polish participants; therefore, the observations should be interpreted with caution when extrapolating the results to other ethnic and geographic populations. A further limitation of the present study is the absence of albuminuria measurements. Consequently, CKD severity could not be fully characterized according to the KDIGO G/A classification. However, as the primary objective of the study was to investigate metabolic and hormonal parameters in patients with CKD, rather than to provide a comprehensive assessment of CKD severity, eGFR was used as the main parameter of renal function. Also, detailed information on CKD etiology and concomitant pharmacological treatment was not systematically collected and could not be included in the analysis; therefore, their potential influence on the assessed parameters cannot be completely excluded.

Another limitation is the cross-sectional design of the study, as no definitive causal relationships can be inferred between circulating OC levels and CKD-related metabolic, hormonal, or nutritional disturbances. A further consideration is that adjustment for multiple comparisons was not performed in the present study, given its exploratory design and the broad range of metabolic, hormonal, and body composition parameters assessed. This should be taken into account when interpreting the observed associations, particularly those with borderline statistical significance. Furthermore, longitudinal assessments of OC levels in individual patients during the progression of CKD and its complications would determine the prognostic value of OC.

## 5. Conclusions

Our observations indicate that circulating OC concentrations are associated with several CKD-related metabolic, nutritional, and hormonal alterations in men with eGFR < 60 mL/min/1.73 m^2^. In particular, serum OC was related to selected parameters reflecting mineral and bone metabolism, as well as metabolic, nutritional, hormonal, anthropometric, and body composition characteristics. These findings suggest that OC may reflect the complex interplay between impaired renal function, alterations in mineral and bone metabolism, and broader systemic metabolic and hormonal changes in CKD. The observed differences between the CKD and control groups and the associations identified in our study may provide further insight into the potential relationship between circulating OC and metabolic and hormonal alterations in CKD. While the present study explored the correlations of OC with a wide range of co-existing CKD alterations, its cross-sectional nature limits the ability to draw causal or prognostic conclusions. However, our findings may serve as a basis for further research exploring these complex relationships and may contribute to a better understanding of the role of OC in metabolic, hormonal, and bone-related disturbances in patients with CKD. Future large-scale prospective studies are required to fully elucidate its prognostic value and potential clinical relevance.

## Figures and Tables

**Table 1:** Clinical characteristics of the studied population.

	Total (n = 144)		The Control Group (n = 46)		The Study Group (n = 98)		*p*-Value
	Mean Median	SD (IQR)	Mean Median	SD (IQR)	Mean Median	SD (IQR)	
Clinical parameters							
Age (years)	62.1 65.5	±13.0 (17.1)	55.7 57.0	±15.8 (26.4)	65.1 67.0	±10.4 (12.5)	**<0.001**
SBP (mmHg)	130.8 130	±15.4 (20)	125.3 130.0	±16.2 19.0	133.5 130.0	±14.5 (15.0)	**0.005**
DBP (mmHg)	77 80	±8.9 (10)	73.6 72.0	±8.7 (10.0)	78.5 80.0	±8.6 (15.0)	**<0.001**
Weight (kg)	88.8 89	±17.2 (21)	87.8 85.2	±18.9 (21.2)	89.3 89.0	±16.4 (21.5)	0.444
BMI (kg/m^2^)	29.1 28.5	±5.1 (7.5)	28.8 28.2	±5.8 (6.4)	29.2 28.7	±4.8 (8.2)	0.389
Body composition parameters							
LTM (kg)	49.5 48	±10.3 (12.7)	47.3 46.7	±10.1 (11.9)	50.8 49.3	±10.3 (13.5)	0.068 *
Rel LTM (%)	56.8 55.9	±12 (18.7)	55.4 54.1	±13.2 (18.7)	57.6 58.5	±11.4 (18.6)	0.314 *
LTI (kg/m^2^)	16.2 15.8	±3 (3.3)	15.5 15.5	±2.9 (2.6)	16.6 16.2	±2.9 (3.5)	0.038 *
Fat (kg)	28.2 26.2	±11.9 (17.8)	29.1 27.7	±13.3 (18.0)	27.8 25.4	±11.0 (16.8)	0.807
Rel fat (%)	30.9 31.7	±8.8 (13.1)	32.1 32.8	±9.5 (12.8)	30.3 30.4	±8.4 (12.6)	0.262 *
FTI (kg/m^2^)	12.6 11.7	±5.3 (7.7)	13.1 12.2	±6.0 (7.4)	12.4 11.2	±4.8 (7.1)	0.117
ATM (kg)	38.4 35.6	±16.1 (24.3)	39.5 37.7	±18.2 (24.6)	37.8 34.6	±15.0 (22.9)	0.799
OH (L)	0.30	±1.7 (1.9)	0.4 0.3	±1.1 (1.6)	0.2 −0.1	±2.0 (2.1)	0.217
Rel OH (%)	1 −0.1	±8.2 (10.3)	1.9 1.7	±5.9 (8.1)	0.5 −0.6	±9.2 (11.5)	0.338 *
TBW (L)	44.4 42.9	±8.4 (9.6)	42.6 41.2	±7.3 (9.7)	45.4 43.7	±8.8 (10.1)	0.063
ECW (L)	20.1 19.7	±3.6 (3.6)	19.5 18.8	±3.5 (3.8)	20.4 20.0	±3.6 (3.8)	0.108
ICW (L)	24.4 23.3	±5.8 (5.7)	23.2 22.8	±4.3 (4.5)	25.0 23.9	±6.5 (6.0)	0.093
E/I	0.8 0.8	±0.1 (0.2)	0.8 0.8	±0.1 (0.2)	0.8 0.8	±0.1 (0.2)	0.662
Laboratory parameters							
Serum creatinine (mg/dL)	1.9 1.6	±1.3 (1.1)	0.9 0.9	±0.2 (0.3)	2.4 1.9	±1.3 (1.3)	**<0.001**
eGFR (mL/min/1.73 m^2^)	55.6 47.8	±32.7 (45.9)	96.7 97.3	±20.1 (34.2)	36.3 37.2	±14.5 (22.9)	**<0.001**
OC (pg/mL)	11,453.2 8261.3	±10,651.9 (7551.5)	7517.0 7231.2	±3094.4 (4195.1)	13,260.6 9278.8	±12,299.1 (8796.8)	**0.002**
Albumin (g/dL)	4.7 4.4	±3.9 (0.4)	4.4 4.5	±0.4 (0.3)	4.9 4.4	±4.8 (0.5)	0.831
CRP (mg/dL)	1 0.3	±2 (0.7)	2.4 1.0	±3.0 (2.8)	0.4 0.2	±0.5 (0.3)	**<0.001**
Hemoglobin (g/dL)	13.7 14	±1.8 (2.6)	14.7 14.8	±1.3 (1.5)	13.2 13.4	±1.9 (2.5)	**<0.001**
Serum glucose (mg/dL)	117.1 97	±57.3 (36)	112.2 97.5	±52.0 (17.0)	119.4 97.0	±59.7 (45.0)	0.928
Insulin (µIU/mL)	19 13.2	±15.6 (21.2)	18.0 11.3	±15.2 (21.5)	19.5 14.1	±15.8 (20.8)	0.452
Peptide-C (ng/mL)	4.6 4	±2.9 (3.9)	3.4 3.0	±2.0 (2.6)	5.2 5.0	±3.1 (3.8)	**<0.001**
HgbA1c (%)	6.1 5.7	±1.3 (1)	5.7 5.4	±1.1 (0.6)	6.2 5.8	±1.4 (1.1)	**0.007**
HOMA-IR	5.7 3.4	±6 (5.9)	5.8 2.4	±7.1 (6.3)	5.6 3.7	±5.5 (5.8)	0.399
Total cholesterol (mg/dL)	182.1 175	±49.4 (66.5)	194.6 194.0	±49.9 (58.0)	176.2 165.0	±48.3 (64.0)	**0.030**
Low-density lipoprotein cholesterol (mg/dL)	115.6 108.1	±43.4 (59.6)	123.6 111.8	±44.4 (61.1)	112.0 103.5	±42.7 (59.0)	0.133
High-density lipoprotein cholesterol (mg/dL)	46 44.1	±13.1 (17.3)	47.0 46.4	±9.7 (13.8)	45.5 42.0	±14.4 (19.0)	0.129
Triglycerides (mg/dL)	174.2 141	±118.4 (97.5)	138.5 126.5	±60.2 (68.0)	190.9 144.5	±134.5 (108.0)	**0.025**
Parathyroid hormone (pg/mL)	80.2 46.2	±98.6 (57.8)	32.1 30.8	±15.6 (17.8)	103.0 64.1	±112.4 (78.8)	**<0.001**
Vitamin D (ng/mL)	22.5 21.1	±10.7 (13.6)	23.0 20.7	±11.0 (14.7)	22.2 21.1	±10.7 (13.9)	0.855

SBP, systolic blood pressure; DBP, diastolic blood pressure; BMI, body mass index; LTM, lean tissue mass; Rel LTM, relative lean tissue mass; LTI, lean tissue index; Fat, fat mass; Rel fat, relative fat mass; FTI, fat tissue index; ATM, adipose tissue mass; OH, overhydration; Rel OH, relative overhydration; TBW, total body water; ECW, extracellular water; ICW, intracellular water; E/I, extracellular water/intracellular water; eGFR, estimated glomerular filtration rate; OC, osteocalcin; CRP, C-reactive protein; HgbA1c, hemoglobin A1c; HOMA-IR, Homeostatic Model Assessment of Insulin Resistance; *p*-values < 0.05 are marked in bold. * Student *t*-test.

**Table 2:** The correlations of osteocalcin with clinical, body composition and laboratory parameters.

	Total (n = 144)		The Control Group (n = 46)		The Study Group (n = 98)	
	r	*p*	r	*p*	r	*p*
Clinical parameters						
Age (years)	−0.082	0.332	−0.079	0.608 *	−0.207	**0.041**
SBP (mmHg)	0.186	**0.028**	−0.083	0.594 *	0.260	**0.011**
DBP (mmHg)	0.076	0.374	−0.214	0.164 *	0.070	0.499
Weight (kg)	−0.190	**0.024**	−0.356	**0.016**	−0.171	0.097
BMI (kg/m^2^)	−0.277	**<0.001**	−0.340	**0.022**	−0.293	**0.004**
Body composition parameters						
LTM (kg)	0.044	0.622	−0.094	0.543 *	0.001	0.992
Rel LTM (%)	0.220	**0.013**	0.118	0.445 *	0.234	**0.033**
LTI (kg/m^2^)	−0.024	0.786	−0.169	0.272 *	−0.076	0.494
Fat (kg)	−0.264	**0.003**	−0.220	0.152	−0.304	**0.005**
Rel fat (%)	−0.248	**0.005**	−0.086	0.579 *	−0.294	**0.007**
FTI (kg/m^2^)	−0.300	**<0.001**	−0.204	0.184	−0.357	**<0.001**
ATM (kg)	−0.264	**0.003**	−0.220	0.152	−0.305	**0.005**
OH (L)	0.116	0.193	−0.284	0.062 *	0.306	**0.005**
Rel OH (%)	0.113	0.205	−0.270	0.076 *	0.309	**0.005**
TBW (L)	−0.061	0.491	−0.237	0.122 *	−0.060	0.586
ECW (L)	−0.094	0.294	−0.377	**0.012**	−0.032	0.769
ICW (L)	−0.033	0.711	−0.154	0.319 *	−0.071	0.520
E/I	−0.045	0.616	−0.185	0.230 *	0.067	0.543
Laboratory parameters						
Serum creatinine (mg/dL)	0.484	**<0.001**	0.116	0.452 *	0.590	**<0.001**
eGFR (mL/min/1.73 m^2^)	−0.473	**<0.001**	−0.029	0.850 *	−0.581	**<0.001**
Albumin (g/dL)	−0.184	**0.029**	0.347	**0.019**	−0.373	**<0.001**
CRP (mg/dL)	−0.013	0.878	−0.033	0.831	0.140	0.170
Hemoglobin (g/dL)	−0.271	**0.001**	−0.158	0.301	−0.314	**0.002**
Serum glucose (mg/dL)	0.107	0.204	−0.005	0.974	0.150	0.143
Insulin (µIU/mL)	−0.247	**0.003**	−0.189	0.214	−0.305	**0.002**
Peptide-C (ng/mL)	0.009	0.914	−0.269	0.075	0.015	0.884
HgbA1c (%)	−0.025	0.764	−0.071	0.648	−0.086	0.400
HOMA-IR	−0.181	**0.031**	−0.185	0.223	−0.220	**0.029**
Total cholesterol (mg/dL)	−0.043	0.609	−0.007	0.968 *	−0.024	0.811
Low-density lipoprotein cholesterol (mg/dL)	−0.020	0.809	0.037	0.238	−0.013	0.896
High-density lipoprotein cholesterol (mg/dL)	0.145	0.083	0.120	0.438 *	0.163	0.109
Triglycerides (mg/dL)	−0.116	0.167	−0.351	**0.018**	−0.120	0.241
Parathyroid hormone (pg/mL)	0.480	**<0.001**	−0.143	0.354	0.555	**<0.001**
Vitamin D (ng/mL)	−0.015	0.857	0.092	0.552	−0.061	0.558

SBP, systolic blood pressure; DBP, diastolic blood pressure; BMI, body mass index; LTM, lean tissue mass; Rel LTM, relative lean tissue mass; LTI, lean tissue index; Fat, fat mass; Rel fat, relative fat mass; FTI, fat tissue index; ATM, adipose tissue mass; OH, overhydration; Rel OH, relative overhydration; TBW, total body water; ECW, extracellular water; ICW, intracellular water; E/I, extracellular water/intracellular water; eGFR, estimated glomerular filtration rate; CRP, C-reactive protein; HgbA1c, hemoglobin A1c; HOMA-IR, Homeostatic Model Assessment of Insulin Resistance; *p*-values < 0.05 are marked in bold. * Pearson correlation test; r—correlation coefficient.

**Table 3:** Significant predictors of osteocalcin levels in univariable regression analysis.

	Total (n = 144)		The Control Group (n = 46)		The Study Group (n = 98)	
	B	*p*	B	*p*	B	*p*
Clinical parameters						
SBP (mmHg)	209.0	**<0.001**			296.6	**<0.001**
Body composition parameters						
Rel LTM (%)	174.8	**0.016**			249.3	**0.026**
Fat (kg)	−202.5	**0.006**			−307.0	**0.008**
Rel fat (%)	−312.8	**0.001**			−462.7	**0.002**
FTI (kg/m^2^)	−495.7	**0.003**			−765.9	**0.003**
ATM (kg)	−148.5	**0.006**			−225.3	**0.008**
OH (L)					2584.3	**<0.001**
Rel OH (%)					552.2	**<0.001**
ECW (L)			−278.3	**0.040**		
Laboratory parameters						
Serum creatinine (mg/dL)	5514.8	**<0.001**			6284.1	**<0.001**
eGFR (mL/min/1.73 m^2^)	−139.4	**<0.001**			−484.7	**<0.001**
Albumin (g/dL)			2854.6	**0.016**		
Hemoglobin (g/dL)	−2323.6	**<0.001**			−2509.2	**<0.001**
Insulin	−2063.92	**0.031**			−2609.6	**0.039**
HOMA-IR	−2063.92	**0.031**			−2609.6	**0.039**
Triglycerides (mg/dL)			−17.6	**0.020**		
Parathyroid hormone (pg/mL)	74.2	**<0.001**			73.9	**<0.001**
Vitamin D					−6219.1	**0.013**

SBP, systolic blood pressure; Rel LTM, relative lean tissue mass; Fat, fat mass; Rel fat, relative fat mass; FTI, fat tissue index; ATM, adipose tissue mass; OH, overhydration; Rel OH, relative overhydration; ECW, extracellular water; eGFR, estimated glomerular filtration rate; HOMA-IR, Homeostatic Model Assessment of Insulin Resistance; *p*-values < 0.05 are marked in bold. B—regression coefficient.

**Table 4:** Multivariable backward regression analysis of osteocalcin.

	Total (n = 144)		The Control Group (n = 46)		The Study Group (n = 98)	
	B	*p*	B	*p*	B	*p*
FTI (kg/m^2^)	−426.6	**0.002**			−625.1	**0.002**
Rel OH (%)					373.4	**0.001**
Albumin (g/dL)			2489.9	**0.034**		
Hemoglobin (g/dL)	−953.3	**<0.001**				
Triglycerides (mg/dL)			−15.3	**0.045**		
Parathyroid hormone (pg/mL)	61.2	**0.022**			66.8	**<0.001**
Model result	R^2^ = 0.418	***p*** **< 0.001**	R^2^ = 0.200	***p*** **= 0.010**	R^2^ = 0.470	***p*** **< 0.001**

FTI, fat tissue index; Rel OH, relative overhydration; *p*-values < 0.05 are marked in bold. B—regression coefficient.

## Data Availability

The data presented in this study are available upon request from the corresponding author. The data are not publicly available due to the Polish General Data Protection Regulation.
